# Towards Understanding Aerogels’ Efficiency for Oil Removal—A Principal Component Analysis Approach

**DOI:** 10.3390/gels9060465

**Published:** 2023-06-06

**Authors:** Khaled Younes, Mayssara Antar, Hamdi Chaouk, Yahya Kharboutly, Omar Mouhtady, Emil Obeid, Eddie Gazo Hanna, Jalal Halwani, Nimer Murshid

**Affiliations:** 1 College of Engineering and Technology, American University of the Middle East, Egaila 54200, Kuwait; mayssara.antar@aum.edu.kw (M.A.); hamdi-chaouk@aum.edu.kw (H.C.); yahya.kharboutly@aum.edu.kw (Y.K.); omar.mouhtady@aum.edu.kw (O.M.); emil.obeid@aum.edu.kw (E.O.); eddie-hanna@aum.edu.kw (E.G.H.); 2 Water and Environment Sciences Laboratory, Lebanese University, Tripoli P.O. Box 6573/14, Lebanon; jhalwani@ul.edu.lb

**Keywords:** aerogel, machine learning, oil removal, principal component analysis, sustainability, unsupervised learning

## Abstract

In this study, our aim was to estimate the adsorption potential of three families of aerogels: nanocellulose (NC), chitosan (CS), and graphene (G) oxide-based aerogels. The emphasized efficiency to seek here concerns oil and organic contaminant removal. In order to achieve this goal, principal component analysis (PCA) was used as a data mining tool. PCA showed hidden patterns that were not possible to seek by the bi-dimensional conventional perspective. In fact, higher total variance was scored in this study compared with previous findings (an increase of nearly 15%). Different approaches and data pre-treatments have provided different findings for PCA. When the whole dataset was taken into consideration, PCA was able to reveal the discrepancy between nanocellulose-based aerogel from one part and chitosan-based and graphene-based aerogels from another part. In order to overcome the bias yielded by the outliers and to probably increase the degree of representativeness, a separation of individuals was adopted. This approach allowed an increase in the total variance of the PCA approach from 64.02% (for the whole dataset) to 69.42% (outliers excluded dataset) and 79.82% (outliers only dataset). This reveals the effectiveness of the followed approach and the high bias yielded from the outliers.

## 1. Introduction

Water scarcity is a growing concern worldwide, affecting many regions and populations. According to the United Nations, more than 2 billion people live in countries experiencing high water stress, and this number is expected to increase in the coming years due to population growth, climate change, and other factors [1]. Water scarcity can have a significant impact on human health, agriculture, and economic development. In many areas, people have to travel long distances to access safe drinking water, which can lead to health problems [1]. To address water scarcity, there are many efforts underway to improve water management and conservation practices. This includes initiatives to promote more efficient water use in agriculture, industry, and households, as well as investments in infrastructure to improve water storage, distribution, and water treatment techniques [2].

There are several novel water treatment techniques that have been developed to address water scarcity and improve water quality. Membrane filtration is a technique that involves using membranes to filter out impurities and contaminants from water [3]. It can be used for desalination, removal of microorganisms and pollutants such as pesticides and pharmaceuticals [3]. Electrocoagulation involves passing an electric current through contaminated water, which causes the contaminants to coagulate and settle down. This technique is effective in removing a range of contaminants, including heavy metals and organic compounds [4]. Membrane distillation involves using a hydrophobic membrane to separate water from contaminants through the process of evaporation and condensation. This technique is particularly effective for removing contaminants that have a high boiling point [5]. Biosorption is a technique that uses living or non-living biomass to remove contaminants from water [6]. It can be effective in removing a wide range of contaminants from water-rich inorganic matter contaminants [6].

Aerogels have potential applications in water treatment due to their unique properties, such as high surface area, low density, and high porosity [2,7,8]. One potential use of aerogels in water treatment is as a filtration medium. The high surface area of aerogels allows them to trap and remove impurities from water [2,7,8]. Aerogels can be used as filter media to remove heavy metals, organic compounds, and other contaminants from water [9,10]. Oil–water separation using aerogels has been intensively studied and evaluated [10]. Another potential application of aerogels in water treatment is as a sorbent material, as it can absorb large amounts of contaminants [7]. Additionally, it can act as a catalyst for the oxidation or reduction of contaminants, giving aerogels the capacity to break down organic compounds in wastewater [11]. Hence, the unique properties of aerogels make them promising materials for water treatment applications [2,7,8]. However, further research is needed to explore their potential applications and optimize their performance in different water treatment scenarios. One way to seek potential uses for these types of membranes is by applying data analysis on the dataset encompassing physico-chemical properties, adsorption parameters, and even manufacturing conditions and trade-offs [12,13]. One of the most suited data analysis techniques is “Principal Component Analysis” (PCA).

PCA is statistical technique used to reduce the dimensionality of a large dataset by identifying patterns and correlations among variables. In other words, it aims to identify the underlying factors that explain the most variance in the data [14]. The basic idea behind PCA is to transform a large dataset into a smaller one by projecting the original data onto a new set of axes, called principal components PCs, that capture most of the variability in the data. The first PC is the direction in which the data varies the most, while the second PC is the direction that captures the most remaining variability, and so on [14]. PCs are actually orthogonal in relation to each other; this represents the geometrical interpretation of no correlation between PCs [14]. PCA is widely used in various fields, such as geology [15,16], biomass characterization, and valorization [17]. For aerogels, our previous studies applied PCA for the sake of revealing hidden patterns between the physical and chemical properties and adsorption parameters [12,13]. The machine learning approach found its importance in evaluating several water treatment processes, including the efficiency of nanofiltration membranes and the fabrication of sustainable materials [18,19,20].

In this study, we have attempted to apply PCA for the dataset of nanocellulose (NC), chitosan (CS), and graphene oxide (G)-based aerogels for the sake of identifying their efficiency towards the removal of oil and organic contaminants from water. PCA allows us to unveil hidden patterns that could not be shown by the bi-dimensional perspective. This will raise two questions, can the accordance/discrepancy of physical and chemical phenomena be explained by PCA? Are there any hidden inquiries that could be shown by PCA and can be explained by chemical and physical intuition?

## 2. Results and Discussion

PCA analysis was conducted and plotted based on previously published data (Table 1) from the study of Ahankari and Paul [2].

**Figure 1:**
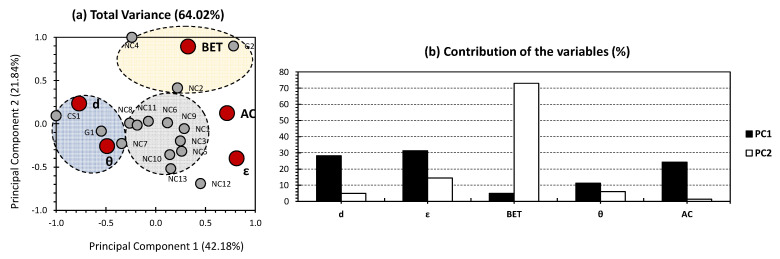
(**a**) PCA biplot representation of all datasets for the oil removal results (data were obtained from the previous investigations of Paul and Ahankari [2]). Small grey bullets present the samples of the population (CS-, G-, and NC-based aerogels). Big red bullets present the factors influencing the samples (physico-chemical properties and adsorption parameters). (**b**) % contribution of the investigated variables towards PC1 (black bars) and PC2 (white bars).

Figure 1 presents the PCA results targeting the previously published investigations of Paul and Ahankari [2]. The individual populations encompass several aerogel types, mostly NC-based aerogels, one CS-based, and two G-based aerogels. The first two PCs exhibited 64.02% of the total variance (42.18% for PC1 and 21.84% for PC2; Figure 1a). Interestingly, higher variance in the case of oil removal was obtained in comparison to the dataset investigated for the case of ion and dye removal [12,13]. This indicates a higher scope of applicability of the adopted method for the sake of revealing the hidden patterns and the certain correlation between physico-chemical properties from one side and adsorption parameters from another side, in the case of oil removal. The aforementioned findings may indicate the presence of some other properties influencing the activity of the investigated aerogels towards oil removal, yet to a lower extent than the case of ion and dye removal [12,13]. For organic molecules, the stipulation of molecular polarity and capacity to produce hydrogen bonds should be taken into consideration, for the sake of better seeking its influence, in regard to the molecular interaction of pollutants with the investigated type of matrices [37].

Density, porosity, and adsorption capacity showed the highest contribution towards PC1, accounting for 28.151%, 31.248%, and 24.28%, respectively (Figure 1b). BET and water contact angle showed a minor contribution towards PC1. As for PC2, the major contribution was scored for BET, accounting for 73% of the total contribution for this axis. Following the orthogonality (which is the geometrical interpretation for the lack of correlation) between PC1 and PC2, the aforementioned findings indicate the strong correlation/influence of the density and porosity as physico-chemical properties towards adsorption capacity. On the other hand, these trends indicate the minor influence of these properties on the adsorption mechanism of oils, represented here by the BET adsorption isotherm. The latter statement makes sense since porosity and density are more likely mechanical properties, which are not likely to influence the electronic/polarity features of the investigated aerogels. In other words, density and porosity influence oil’s removal process at the macroscopic/mesoscopic scale. In order to better understand the microscopic behavior and adsorption mechanisms, a more sophisticated dataset should be taken into consideration. On the other hand, enlarging the dataset will possibly create a bias in the trends, and several unexplainable features could arise.

**Figure 2:**
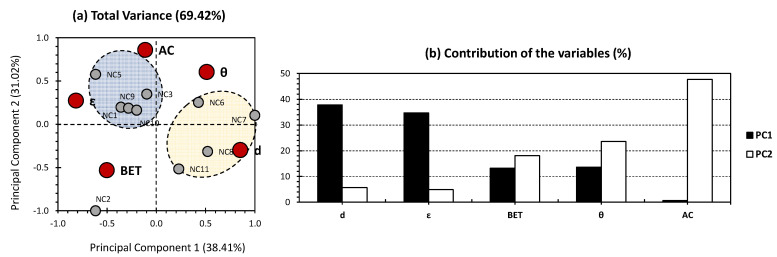
(**a**) PCA biplot representation of the reduced dataset for the oil removal results (data were obtained from the previous investigations of Paul and Ahankari [2]; CS1, G1, G2, NC4, NC12, and NC13 were excluded). Small grey bullets present the samples of the population (CS-, G-, and NC-based aerogels). Big red bullets present the factors influencing the samples (physico-chemical properties and adsorption parameters). (**b**) % contribution of the investigated variables towards PC1 (black bars) and PC2 (white bars).

**Figure 3:**
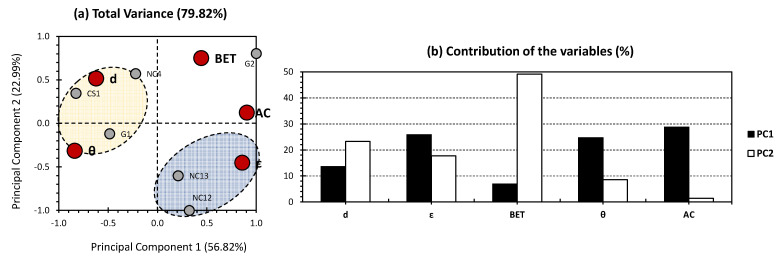
(**a**) PCA biplot representation of the excluded dataset from Figure 2 (data were obtained from the previous investigations of Paul and Ahankari [2]; CS1, G1, G2, NC4, NC12, and NC13 were exclusively included). Small grey bullets present the samples of the population (CS-, G-, and NC-based aerogels). Big red bullets present the factors influencing the samples (physico-chemical properties and adsorption parameters). (**b**) % contribution of the investigated variables towards PC1 (black bars) and PC2 (white bars).

For the individuals, three different clusters were observed (grey, blue, and yellow; Figure 1a). Interestingly, most of the investigated samples were located in the middle of the bi-plot (shown in the grey cluster). These trends could arise from several possibilities: (1) the skewing effect of most influencing variables (CS1, G1, G2, NC4, NC12, and NC13, in our case). This could yield some bias in the different trends of the other samples. (2) The low influence of the other samples (samples of the grey cluster). This could be due to the fact that these variables are more likely similar to each other than the samples of the blue and yellow cluster, and follow certain “conventional” oil decontamination trends, which were missing in the excluded samples. In order to confirm or deny these two hypotheses, two separate PCA investigations were conducted: one for the whole dataset with the exclusion of the samples with the major influence (Figure 2) and another for exclusively describing these samples (Figure 3). Nonetheless, several patterns can be explained in Figure 1, even though a certain bias exists. The blue cluster arranges aerogels CS1, G1, and NC7 and shows a high positive correlation concerning density and water contact angle. This probably indicates that these types of aerogels are more likely to be applied in the case of higher-density membranes, and more water contact angles are to be implemented. These conditions are applied in the case of highly corrosive media. For the yellow cluster, including NC2, NC4, and G2 aerogels, a high positive correlation along BET was shown. Interestingly, the latter parameter was the major contributor of the second PC, indicating that the aerogels of this cluster possess the same status as BET in the sense that they are not dramatically influenced by density and porosity as physical properties and are also more likely independent of other physical and adsorption parameters. The grey cluster gathered most of the investigated samples. It is quite interesting that exclusively NC-based aerogels were gathered in this cluster (Figure 1a). The different samples were located in the middle of the PCA bi-plot and showed no proximity to any of the investigated variables. This indicates the equal distributed effect of the variables on the different NC aerogels under consideration.

Figure 2 presents the PCA results targeting the previously published investigations of Paul and Ahankari [2], excluding the factors presenting the highest variance (CS1, G1, G2, NC4, NC12, and NC13). The first two PCs exhibited 69.42% of the total variance (38.41% for PC1 and 31.02% for PC2; Figure 2a). Interestingly, moderately higher variance was obtained in comparison with the all-dataset approach. This indicates the efficiency of excluding outliers, as they contribute to a sort of bias for the dataset. This increase in variance reveals better decision-making for the different trends of this PCA’s bi-plot. Nonetheless, more physico-chemical propertiesneed to be taken into account for the sake of revealing the full picture in regard to the differences between the investigated aerogels. On the other hand, it is worth mentioning that the difference in variance between PC1 and PC2 was less than the one existing between the first two PCs of the whole-dataset approach (Figure 1).

For the variables, only the density and porosity showed the highest contribution towards PC1, accounting for 37.81% and 34.70%, respectively (Figure 2b). BET and water contact angle showed moderate contribution towards both PCs. As for PC2, the major contribution was scored for adsorption capacity, accounting for 47.69% of the total contribution for this axis. The aforementioned findings indicate a minor correlation/influence of the density and porosity as physico-chemical properties towards adsorption capacity. Interestingly, opposite findings were obtained in the case of the whole-dataset approach. This reveals the interest and strength of applying separation of individuals in order to eliminate any possible discrepancy due to their high weightage. Since higher variance was yielded in this case, more reliability can be given to its trends, rather than one of the whole datasets being considered (Figure 1 and Figure 2). For the individuals, two clusters were obtained (blue and yellow clusters). The blue cluster arranged aerogels NC1, NC3, NC5, NC9, and NC10, showing a high positive correlation between porosity and adsorption capacity. This probably indicates that these types of aerogels are more likely to be applied in cases where a high porosity is required, and higher purity requirements of water are envisaged since a higher adsorption capacity is required. The yellow cluster included NC6, NC7, NC8, and NC11 aerogels and showed a moderate to high positive correlation concerning density, indicating that clustered aerogels can be applied in such conditions where a high density of treating material is required.

Figure 3 presents the PCA results exclusively targeting aerogels CS1, G1, G2, NC4, NC12, and NC13, which were excluded from the previous investigation (Figure 2). The first two PCs exhibited 79.82% of the total variance (56.82% for PC1 and 22.99% for PC2; Figure 3a). This shows the highest total variance among the three different investigations, indicating the highest efficiency of the approach in hand. Therefore, the first hypothesis targeting the skewing effect of the outliers is more likely to be the reason for the bias yielded in the PCA of Figure 1.

Concerning the variables, the contributions towards PC1 were almost equally distributed between porosity, water contact angle, and adsorption capacity, accounting for 25.92%, 24.76%, and 28.80%, respectively (Figure 3b). As for PC2, the major contribution was scored for BET surface area, accounting for 49.10% of the total contribution for this axis. The aforementioned findings indicate a high influence of porosity and water contact angle, as physico-chemical properties, on the adsorption capacity of the investigated aerogels. On the other hand, a minor influence can be noticed for these properties towards the BET surface area. For the individuals, two clusters were obtained (blue and yellow clusters). The blue cluster contained aerogels NC12 and NC13 and showed a high positive correlation for porosity. For the yellow cluster, it included G1 and CS1 and showed a high positive correlation regarding density and water contact angle. Interestingly, the followed approach allowed us to separate NC-based aerogels from one side and CS-and G-based aerogels from another side (Figure 3a).

For the individuals in all three adopted approaches, it is worth mentioning that each approach provided a distinctive pattern of distribution. This indicates that PCA is strictly dependent on the factors and variables being engaged. In other words, if one row of the dataset is removed, a drastic shift in the different patterns will be noticed. Hence, great attention should be taken into consideration when selecting the individuals/factors to be acquired. This should be executed based on the solid knowledge of the user towards the physical and chemical phenomena undergone. PCA works on removing the correlation of a dataset by creating the new uncorrelated variables (PCs). Therefore, the variables should influence one another. Herein, the investigated variables are dependent since they are all factors for estimating the adsorption capacity and mechanism. The significance of the adopted method relies upon the reveal of hidden patterns that could show some intercorrelation between two variables that are usually considered unrelated or hard to grasp a connection between, based on the “physical” and “chemical” intuitions.

## 3. Conclusions

In this study, we attempted to apply the so-called “Principal Component Analysis” (PCA) method for the sake of enhancing our understanding towards the efficiency of mostly nanocellulose, along with chitosan and graphene oxide-based aerogels, for oil and organic pollutants removal. The application of PCA has given a higher representativeness for oil removal compared with the previous findings of dye and ion removal [12,13]. This has been shown by a higher total variance yielded by the PCA of this study. Even though several outliers were yielded when the whole dataset was taken into consideration, the PCA approach was able to decipher the discrepancy between nanocellulose-based aerogel from one side and chitosan-based and graphene-based aerogels from another side. In addition, the whole dataset approach was able to reveal the strong correlation/influence of the density and porosity as physico-chemical properties towards the adsorption capacity of oils. On the other hand, it showed the minor influence of these properties towards the adsorption mechanism of oils. In order to overcome the bias yielded by the outliers and to probably increase the degree of representativeness of the investigated dataset, separation of individuals of the dataset was adopted. This approach allowed an increase in the total variance of the PCA approach from 64.02% (for the whole dataset) to 69.42% (outliers excluded dataset) and 79.82% (outliers only dataset). This shows the effectiveness of the followed approach and the high bias yielded from the outliers.

## 4. Methodology

### 4.1. Data Collection and Pre-Treatment

Physical and chemical features of oil removal’s efficiency were compiled from the published findings of Paul and Ahankari [2]. The list of the various investigated NC-, CS-, and G-based aerogels is shown in Table 1, along with information on their performance potential, adsorption parameters, and physical and chemical properties.

Each investigated variable’s data component has a different weight. To remove any bias caused by the difference of magnitude, a normalization technique similar to that used by Younes et al. [17,38] was implemented as follows:

(1) $$Y_{st}=\frac{\left(Value\;-\;Mean\right)}{Standard\;Deviation},$$

where “*Y_st_*” presents the standardized dataset values.

In this study, the missing data were estimated using a built-in option that replaces any missed value with the “Mode”, following each of the investigated physico-chemical and adsorption properties.

### 4.2. Principal Component Analysis (PCA)

PCA seeks hidden layers between physical and chemical properties on one side and adsorption parameters on the other. If this occurs, it allows for better interpretation and understanding of various factors that influence the applicability of a specific aerogel membrane, in this case, for oil removal.

PCA can provide valuable insights at multiple stages of the water treatment process, from manufacturing methods and experimental conditions to the removal effectiveness of selected membranes. It provides information that can be used to improve the overall efficiency of the water-treatment process. In this study, we used PCA to investigate the influence of eight factors on 24 aerogel samples (Table 1). As an unsupervised machine-learning technique, PCA limits the dimensionality of datasets; this serves to increase data visualization and to seek concealed trends via correlations, either positive or negative. The principal components (PCs) are represented by these new correlations.

PCs present new uncorrelated variables that are compiled by the combination of the different variables. They present orthogonal axes (meaning that they are independent of each other). PCs are ordered in the sense that the first PC (PC1) captures the highest representativeness of the data (highest variance), followed by the second (PC2), capturing the highest variance, and so on. A unit-weighting vector (*Uj*) and the original data matrix *M* with m × n dimensions are used to present the *j*th PC matrix (*Pci*). [6,39,40,41]:

(2) $$Pci=U_{j}^{T}M=\sum_{}^{i=0}U_{ji}M_{i}.$$

In Equation (2), the loading coefficient is represented by *U*, where *M* is the data vector of size *n*. In Equation (3) below, the variance matrix *M*(*Var*(*M*)), which is obtained through the projection of *M* to *U*, should be maximized, following:

(3) $$Var\left(M\right)=\frac{1}{n}\left(UM\right){\left(UM\right)}^{T}=\frac{1}{n}{UMM}^{T}U,$$

(4) $$MaxVar\left(M\right)=Max\left(\left(\frac{1}{n}\right){UMM}^{T}U\right).$$

*Var*(*M*) can be expressed in the following Equation (5) as $\frac{1}{n}{MM}^{T}$ and is the same as the covariance matrix of *M*(*cov*(*M*)):

(5) $$Var\left(M\right)=U^{T}cov\left(M\right)U.$$

By performing the Lagrange multiplier method, the Lagrangian function can be defined as per the following:

(6) $$L=U^{T},$$

(7) $$L=U^{T}cov\left(M\right)U-\delta \left(U^{T}U-1\right).$$

As the weighting vector is a unit vector, “*U^T^U* − 1” is considered equal to zero in Equation (7). Therefore, by equating the derivative of the Lagrangian function (*L*) with respect to *U*, the maximum value of *var*(*M*) can be calculated following:

(8) $$\frac{dL}{dU}=0,$$

(9) $$cov\left(M\right)U-\delta U=\left(cov\left(M\right)-\delta I\right)U=0,$$

where

*δ*: eigenvalue of *cov*(*M*);
*U*: eigenvector of *cov*(*M*).

From an application point of view of PCA, the higher percentage contribution of a variable in a PC indicates the highest efficiency and its greater influence on this PC. Since, in a PCA study, the first two PCs are the most important (having the highest variance), the factors of the original data influencing them the most are the primordial factors, which should be closely regarded and optimized.

## Figures and Tables

**Table 1:** Nanocellulose (NC), chitosan (CS), and graphene (G) oxide-based aerogels in oil removal; physico-chemical properties and adsorption parameters (adapted from Paul and Ahankari [2], copyright (2023), with permission from Elsevier).

NC-Based Aerogels	Aerogel Composition	Physico-Chemical Properties			Adsorption Parameters		Ref
		Density “d” (mg/cm^3^)	Porosity “ε” (%)	Water Contact Angle “θ” (°)	BET Surface Area (m^2^/g)	Adsorption Capacity “AC” (mg/g)	
Nanocellulose (NC)-based Aerogels							
NC1	rGO/MWCNTs-NH2/NC	5.8	-	114.6	176.7	161.7	[21]
NC2	NCA/OA/Fe3O4	9.2	-	84.5	397.5	56.3	[22]
NC3	NC/NCS/rGO	9.3	99	115.3	80.4	171.8	[23]
NC4	MNCAs	9	97	152	841	81	[24]
NC5	Si-CNF/BTCA	6.05	99.6	151	-	151.5	[25]
NC6	Fe3O4/NC	16.7	98.8	146	-	176	[26]
NC7	CNCs/PVA/TEOS	17	98.4	154.9	76	118.5	[27]
NC8	MCPGA	17.95	98.8	142	-	78	[28]
NC9	P-CNS	10.65	99.15	151	362.7	162.5	[29]
NC10	NC/	5.1	99	-	124	108	[30]
NC11	CNCA	16	99	130	-	41.5	[31]
NC12	KNFs	2.7	99.8	150.5	15.85	223	[32]
NC13	HB	7.6	99.5	135	39	107.5	[33]
Chitosan (CS)-based Aerogels							
CS1	silylated CS	27.1	96.8	152.3	20.6	46.5	[34]
Graphene Oxide (G)-based Aerogels							
G1	SGA	14.4	96.9	153	18.5	115	[35]
G2	MCNS/NGA	12	99.5	117.9	787.9	362	[36]

## Data Availability

All data are included in the manuscript.
